# Accuracy of different approaches for detecting proximal root caries lesions in vitro

**DOI:** 10.1007/s00784-022-04709-1

**Published:** 2022-09-16

**Authors:** Gerd Göstemeyer, Mareike Preus, Karim Elhennawy, Falk Schwendicke, Sebastian Paris, Haitham Askar

**Affiliations:** 1 Department of Operative, Preventive and Pediatric Dentistry, Charité – Universitätsmedizin Berlin, Assmannshauser Straße 4-6, 14197 Berlin, Germany; 2 Department of Orthodontics, Dentofacial Orthopedics and Pedodontics, Charité - Universitätsmedizin Berlin, Assmannshauser Straße 4-6, 14197 Berlin, Germany; 3 Department of Oral Diagnostics, Digital Health and Health Services Research, Charité – Universitätsmedizin Berlin, Assmannshauser Straße 4-6, 14197 Berlin, Germany

**Keywords:** Root caries, Geriatric dentistry, Caries diagnosis, Proximal caries, Laser-fluorescence, Near-infrared transillumination

## Abstract

**Objectives:**

The objective was to evaluate the diagnostic accuracy of radiographic evaluation (XR), visual-tactile assessment (VT), laser-fluorescence (LF) (DIAGNOdent Pen/KaVo), and near-infrared-light transillumination (NILT) (DIAGNOcam/KaVo) on proximal root caries lesions in vitro.

**Methods:**

Two-hundred extracted permanent premolars and molars with and without proximal root caries lesions were allocated to 50 diagnostic models simulating the proximal contacts between teeth and mounted in a phantom dummy head. Two independent examiners used the diagnostic approaches to detect any or advanced root caries lesions, with histologic evaluation of the lesions serving as reference. Receiver operating characteristic (ROC) curves were employed, and sensitivity, specificity, and the area under the ROC curve (AUC) are calculated. Significant differences in mean AUCs between approaches were assumed if *p* < 0.05 (two-sample *t*-test).

**Results:**

NILT was not applicable for proximal root caries detection. The sensitivity/specificity to detect any lesions was 0.81/0.63 for XR, 0.76/0.88 for VT and 0.81/0.95 for LF, and the sensitivity/specificity to detect advanced lesions was 0.43/0.94 for XR, 0.66/0.99 for VT, and 0.83/0.78 for LF, respectively. For both, any and advanced root caries lesions, mean AUCs for LF and VT were significantly higher compared to XR (*p* < 0.05). For any root caries lesions, LF was significantly more accurate than VT (*p* = 0.01), but not for advanced root caries lesions (*p* = 0.59).

**Conclusions:**

Under the in vitro conditions chosen, LF and VT were more accurate than XR to detect proximal root caries lesions, with LF being particularly useful for initial lesion stages.

**Clinical relevance:**

LF might be a useful diagnostic aid for proximal root caries diagnosis. Clinical studies are necessary to corroborate the findings.

**Supplementary Information:**

The online version contains supplementary material available at 10.1007/s00784-022-04709-1.

## Introduction

Although there is a significant decline in caries incidence among younger individuals, dental caries remains the most prevalent chronic disease in adults, affecting 2.4 billion individuals worldwide [1]. The overall caries burden has merely shifted from younger to older population groups, as natural teeth that are nowadays retained until older ages, are becoming increasingly susceptible for caries formation [2]. Particularly, the root-caries prevalence and incidence in older patients has substantially increased during the past decades and, taking into account the demographic change, is expected to further dramatically increase in the coming years [2, 3].

Management of root caries lesions can be challenging: placement of restorations is often hampered by difficulties in moisture control, lacking retention to dentin and limited assess if lesions are located proximally. These challenges increase substantially in older patients with limited mobility who are no longer fully capable of restorative dental treatment [4, 5]. A number of alternative treatment strategies other than the restorative approach are available for the treatment of early root caries lesions [6]. However, the correct indication is an important prerequisite for their successful application. Therefore, the application of accurate diagnostic procedures is an important basis for the choice of the best possible management strategy for root caries lesions.

A number of diagnostic approaches are available to assist clinicians in caries detection and diagnosis [7, 8]. Studies evaluating the accuracy of these approaches, however, primarily focus on coronally located caries lesions. Evidence for root caries lesions is limited and does not allow for evaluation of the comparative accuracy between a number of different test methods [9]. Available clinical studies investigating the accuracy of caries diagnostic approaches for root caries diagnosis have compared caries diagnostic devices on easily accessible lesions with visual tactile (VT) examination as a reference test [10, 11]. Particularly for proximal root caries lesions, it is useful to be able to evaluate also the accuracy of VT assessment itself with a histological evaluation of the lesions as reference test, which can be realized mainly under in vitro conditions.

Therefore, the aim of this study was to compare the accuracy of different caries available diagnostic approaches, namely the visual-tactile (VT), radiographic (XR), laser-fluorescence (LF), and near-infrared-light transillumination (NILT) assessment in vitro. The null hypothesis was that there would be no difference in diagnostic accuracy between the different diagnostic methods.

## Materials and methods

### Study design

This study was conducted following an experimental set-up established in our working group for the evaluation of secondary caries lesions in vitro [12]. Four different diagnostic approaches were applied for the detection and evaluation of the proximal root caries lesions:

i. Radiographic evaluation (XR) using digital bitewing radiographs
ii. Visual-tactile assessment (VT) according to the International Caries Detection and Assessment System (ICDAS) [13]
iii. Laser-fluorescence (LF) using DIAGNOdent Pen (KaVo, Biberach, Germany)
iv. Near-infrared-light transillumination (NILT) using DIAGNOcam (KaVo, Biberach, Germany)

**Fig. 1:**
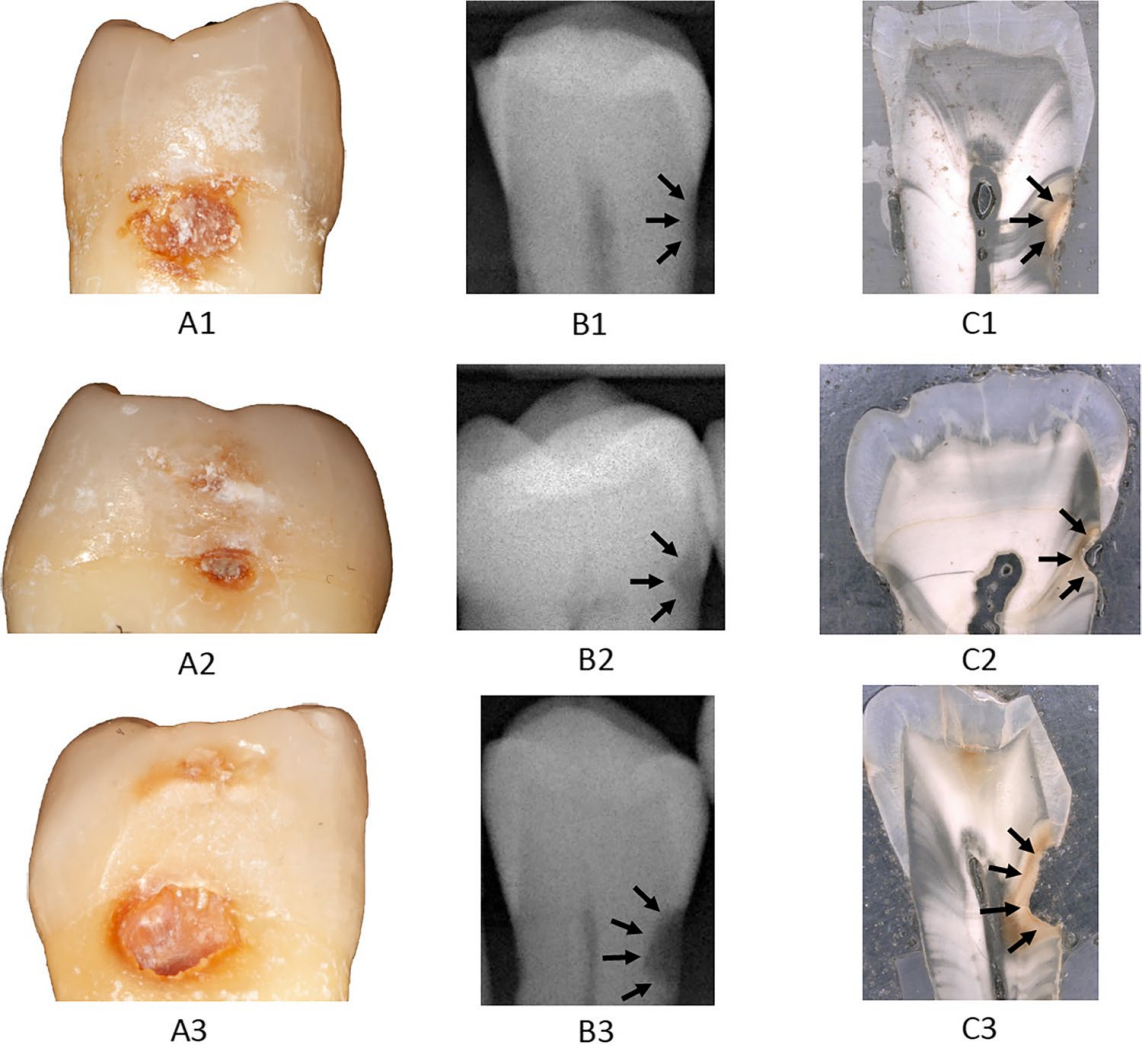
Clinical (**A**), radiographic (**B**) and histological (**C**) images of exemplary teeth (1–3)*.* Black arrows indicate root caries lesions on the radiographs and thin sections

Histological evaluation of the lesions on thin sections was used as a reference. In order to facilitate comparability between the different test methods, their outcome parameters were assigned to three thresholds: (a) no caries, (b) initial root caries lesions, (c) advanced root caries lesions. The criteria used for interpretation of the outcome parameters are shown in Table 1. Exemplary images of included teeth are shown in Fig. 1.

**Table 1:** Criteria for interpretation of the different test methods (radiographic examination: XR, visual-tactile assessment: VT, laser-fluorescence: LF, near-infrared-light transillumination: NILT)

	Interpretation		
Test method	No lesion	Initial lesion	Advanced lesion
XR	No translucency	Translucency with or without cavitation ≤ 0.5 mm	Cavitation > 0.5 mm
VT	No discoloration and no cavitation	Discoloration with or without cavitation ≤ 0.5 mm	Cavitation > 0.5 mm
LF	DIAGNOdent value ≤ 7	DIAGNOdent value 8–15	DIAGNOdent value > 15
NILT	No shadow	Shadow ≤ 0.5 mm	Shadow > 0.5 mm
Reference test	No Demineralisation	Demineralisation with or without cavitation ≤ 0.5 mm	Cavitation > 0.5 mm

### Preparation of diagnostic models

A total of 220 human extracted posterior teeth (110 premolars and 110 molars) that exhibited proximal root caries lesions (157) or not (63) were obtained with informed consent under an ethics-approved protocol (EA4/102/14). Teeth with fractures or deep cracks, crowns, or restorations on root surfaces were excluded. The teeth were continuously stored in 0.5% chloramine-T solution after extraction. Before further use, the teeth were rinsed with water, freed from calculus and debris using a scaler (S1296, Hu-Friedy, Chicago, IL, USA), and cleaned using polishing brushes (ORBIS ZR brushes, nylon bristles, ORBIS Dental, Münster, Germany) and polishing paste (Proxyt, Ivoclar Vivadent, Schaan, Lichtenstein, Germany). Digital photographs (EOS 400d camera, Canon, Tokyo, Japan/90 mm macro lens, Tamron, Saitama, Japan) were taken from all proximal caries lesions. The teeth were then randomly divided into 55 groups of four teeth (two premolars/two molars) each, taking into account the tooth morphology to create realistic approximal spaces. The roots of all teeth were embedded in transparent epoxy resin (Epo-Thin 2, Buehler, Lake Bluff, IL, USA) using a silicone mold, with tight proximal contacts ensured between neighboring teeth. After setting of the epoxy resin, the shape of the gingiva was modeled with pink wax (S-U- Plattenwachs rosa, Schuler-Dental, Ulm, Germany), whereby the root surfaces to be diagnosed were not covered by wax (Appendix Fig. S1). The resulting 55 diagnostic models (50 models for the main evaluation, 5 models for calibration) were stored in 0.5% chloramine-T solution until further usage.

### Preparation of radiographs

The study models were mounted in a custom-made device, allowing for orthograde radiographs being taken. The source-to-film distance was 250 mm. A 15-mm-thick plexiglass plate was placed between the source and the model to stimulate soft tissue scattering [14]. Digital bitewing radiographs were taken of all groups of 4 teeth in the study models using a radiation source (Heliodent Plus, Sirona Dental Systems, Bensheim, Germany) and a sensor (XIOS XG, Sirona Dental Systems, Bensheim, Germany), operating at 65 kV and 7 mA with an exposure time of 0.06 s.

### Index tests

Only the proximal surfaces with adjacent teeth were evaluated, resulting in 6 test surfaces per model. XR evaluation was performed on a diagnostic screen. VT, LF, and NILT tests were performed on the study models mounted in a phantom dummy head (Phantomkopf PK-2 TSE St, Gesichtsmaske P-6 GM, Frasaco, Tettnang, Germany). The study models were mounted alternately in the upper or lower jaw position.

Two dentists with several years of clinical experience (GG, HA) performed caries assessment using the different diagnostic methods. Prior to each test, the examiners were provided with theoretical information about each test method and calibrated using 5 exemplary models. Only one diagnostic method was used in each test session. A minimum interval of 2 weeks was maintained between each test session.

#### Radiographic assessment

The digital radiographs were assessed on a 27-inch diagnostic screen using a diagnostic software (Sidexis XG, Sirona Dental Systems, Bensheim, Germany). No modification of the original image or screen adjustments was allowed and no image enhancement software was used. The radiographs were scored as no translucency, translucency ≤ 0.5 mm in the direction of the pulp and translucency > 0.5 mm (Table 1).

#### Visual-tactile assessment

VT was performed under the operating light of a dental chair (KaVoLUX 540 LED, KaVo, Biberach, Gernany) using magnifying glasses with 2.5× magnification, a dental mirror (M4C, Hu-Friedy, Chicago, IL, USA), a dental probe (1085/9, Henry Schein, Melville, USA), and a 3-way air syringe. The proximal root surfaces were carefully palpated with the probe without pressure. Carious lesions were suspected if any irregularities or gaps were detected by visual inspection and probing. The results of the VT were graded according to the ICDAS criteria as follows [13]: as no irregularities or gap, irregularities or gap ≤ 0.5 mm, and gap > 0.5 mm.

#### Laser-fluorescence

LF was performed using a DIAGNOdent pen device (KaVo, Biberach, Germany). To minimize interference with external light sources, direct light exposure from the operating light was avoided during the measurement. Prior to each test session, the DIAGNOdent pen was calibrated according to the manufacturer’s instructions using the corresponding ceramic standard and the probe tip was checked for its integrity. The DIAGNOdent pen was calibrated to the background fluorescence of each tooth to be examined at a caries-free site. By repeated pressure-free probing, the tip of the probe was inserted into the proximal spaces from the buccal and oral sides, and the maximum measured value (peak value) was determined. The measurement was repeated three times per examined tooth surface and the mean value of the three measurements was determined before data analysis. The classification according to the severity of the caries lesions was made in accordance with the manufacturer’s instructions for approximal caries: values ≤ 7 (no caries), values 8–15 (incipient demineralization) and values > 15 (distinct demineralization).

#### Near-infrared transillumination

NILT was performed a DIAGNOcam device (KaVo, Biberach, Germany) and associated software (KaVo KiDSoftware, KaVo Integrated Desktop/Version 2.4.1.6821, Biberach, Germany), which was run on a tablet PC (Microsoft Surface Pro2, Redmond, USA). The operating light was switched off during the measurement to avoid interferences with the camera. The examination was performed on moisturized teeth. The camera unit was moved perpendicularly to the occlusal surfaces of the examined teeth. To simulate the clinical use of the device, no images were taken, but the teeth were assessed by the examiners live and in motion of the camera.

### Reference test

As a reference, the extent of the caries lesions was evaluated on tooth sections under a digital light microscope. The methodology of evaluation was described by Karlsson et al. [15]. The teeth were removed from the diagnostic models and cut along the mesio-distal direction through the region with the greatest coronal-apical extension on the surface of the caries lesion using a diamond band saw (Band Saw Exakt 300 CL, Exakt Apparatebau, Norderstedt, Germany) with a 0.2-mm-thick saw blade. The tooth sections to analyze were then embedded into polymethylmethacrylate (Technovit 4004, Kulzer, Hanau Germany) and the cut surfaces with the caries lesions were ground flat (Phoenix Alpha, Buehler, Düsseldorf, Germany) and polished (SiC paper 1200, 2400, 4000 Exakt Apparatebau) until slices of 300 μm thickness were obtained.

The extent of the caries lesions on the thin sections was evaluated under a digital light-microscope at 20 × magnification (VHX-5000, Keyence, Osaka, Japan) according to a method previously reported [15]. A straight line was drawn along the suspected original root surface (Appendix Fig S2). Cavity depth was determined along a second line, which was drawn perpendicular from the first line to the deepest point of the cavity. In order to allow comparison with the index tests, the values were assigned to two different cutoff points: (a) caries with a cavity depth of 0–0.5 mm and (b) caries with a cavity depth > 0.5 mm.

### Reliability

Inter-examiner reliability was assessed by determination of the agreement of the results for each diagnostic methods from the main tests. Intra-examiner reliability was evaluated assessed by repeated examination of the surfaces of 5 additional diagnostic models (30 root surfaces) by both examiners, separated by a 2-week interval. Reliability was calculated as kappa (κ)-values and interpreted according to the classification of Landis and Koch [16].

### Statistical analysis

The diagnostic value of the different test methods was evaluated for two different cutoff points: (1) detection of any lesions (i.e., initial and advanced lesions) and (2) detection of advanced lesions only. Sensitivity, specificity, positive (PPV), and negative predictive values (NPV) were calculated. Receiver-operating characteristics (ROC) curves were employed and the resulting area under the curve (AUC) was calculated to quantify the diagnostic value of each test. Differences in AUC values of each test were calculated using two-sample *t*-test with the level of significance of *p* = 0.05. The statistical evaluation was performed with SPSS 22 (IBM, Armonk, USA).

## Results

Six carious tooth surfaces were excluded from the study because the specimens were lost during preparation of the tooth sections for the reference test, leaving 149 carious and 145 non-carious root surfaces available for analysis. Application of NILT did not allow an adequate diagnosis of proximal root caries lesions. The interdental spaces were overexposed, and therefore, it was not possible to assess the root surface in any measurement (Appendix Fig. S3). All other diagnostic methods could be applied.

**Fig. 2:**
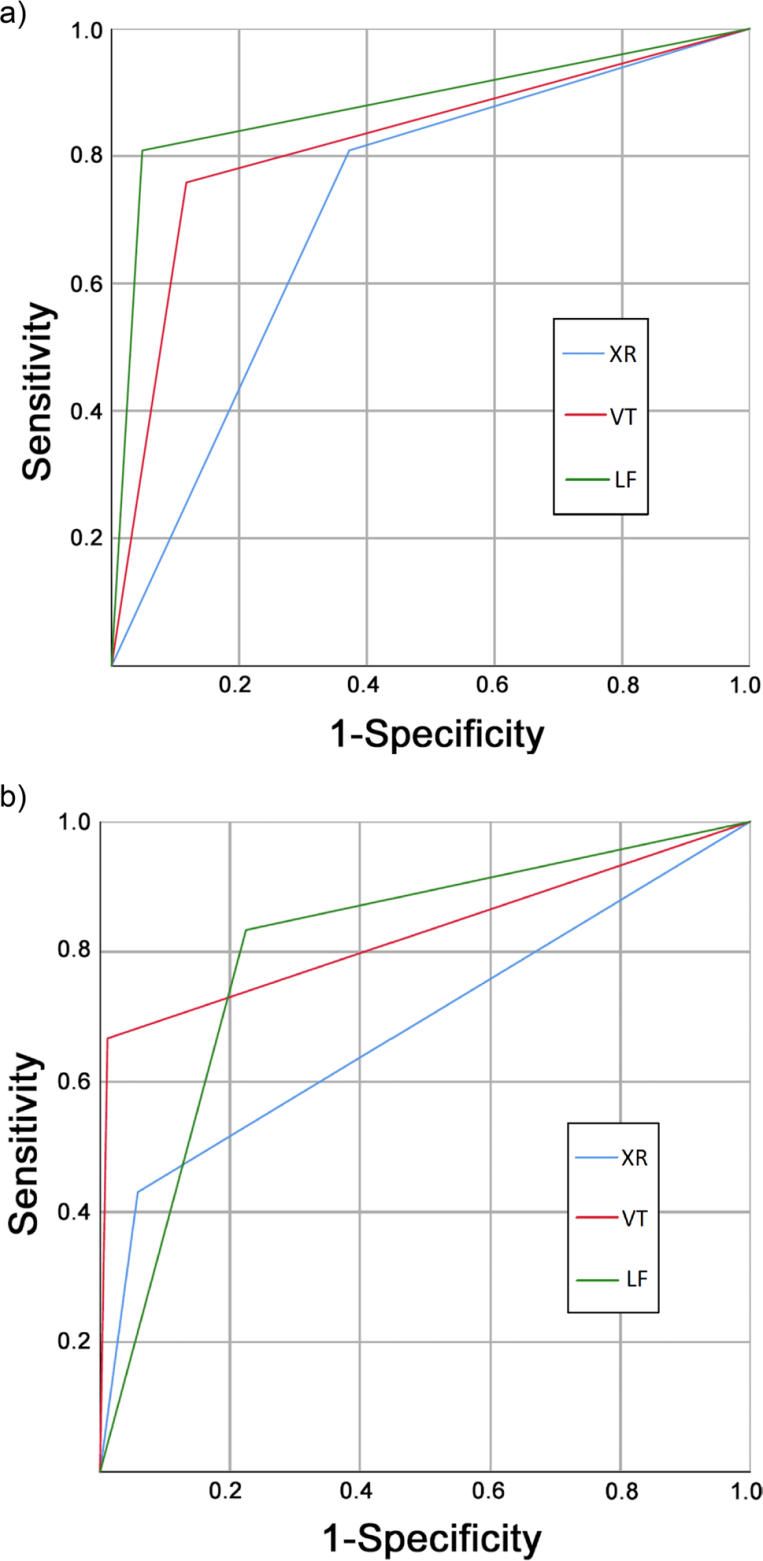
Receiver operating characteristics (ROC) curves for radiographic (XR), visual-tactile (VT), and laser-fluorescence (LF) methods for the detection of any (**a**) and advanced (**b**) lesions. The diagnostic value of each test method was calculated as the area under the curves (AUC) from the respective ROC curves (AUC values are given in Table 2)

For detection of any root caries lesions, XR und LF had the highest sensitivity (both 0.81) with LF also having the highest specificity (0.95) (Table 2, Fig. 2). The AUC value of LF (0.88) was significantly higher compared to the other diagnostic methods (*p* < 0.05/ two-sample *t*-test) (Table 3). For the detection of advanced root caries lesions, LF had the highest sensitivity (0.83) but the lowest specificity (0.78). The AUC values of VT (0.83) and LF (0.80) were significantly higher than XR (*p* < 0.05) with no significant difference between VT and LF (*p* = 0.59).

**Table 2:** Sensitivity (Sens) specificity (Spec), area under the curve (AUC: calculated from the ROC curves in Fig. 2), positive (PPV) and negative (NPV) predictive values of radiographic (XR), visual-tactile (VT), and laser-fluorescence (LF) assessment methods for the detection of any lesions and advanced lesions

	Any lesions (*prevalence 50.6%*)					Advanced lesions (*prevalence 12.2%*)				
Test method	Sens	Spec	AUC	PPV	NPV	Sens	Spec	AUC	PPV	NPV
XR	0.81	0.63	0.72	0.68	0.77	0.43	0.94	0.69	0.50	0.92
VT	0.76	0.88	0.82	0.87	0.79	0.67	0.99	0.83	0.88	0.96
LF	0.81	0.95	0.88	0.94	0.84	0.83	0.78	0.80	0.34	0.97

**Table 3:** Comparison of the diagnostic quality (according to area under the curve (AUC) values) between the different assessment methods. Mean differences (MD), standard errors (SE), and *p*-values are given for the comparisons between the different assessment methods (radiographic examination (XR), visual-tactile assessment (VT), and laser-fluorescence (LF)). Comparisons which differ significantly (*p* < 0.05, two-sample *t*-test) are indicated with an asterisk (*)

	Any lesions			Advanced lesions		
	MD	SE	*p-*value	MD	SE	*p*-value
XR/VT	0.103	0.028	0.0002*	0.142	0.052	0.0059*
XR/LF	0.162	0.026	< 0.0001*	0.118	0.048	0.0146*
LF/VT	0.059	0.023	0.0118*	0.024	0.044	0.586

The intra-rater reliability for detection of any root caries lesions was perfect for XR (*κ* = 1.0), while LF and VT had substantial agreement (Table 4). The inter-rater reliability was moderate for XR (*κ* = 0.42) and substantial for LF and VT. The intra-rater reliability for detection of advanced lesions was substantial for VT and XR and moderate for LF (*κ* = 0.56). The inter-rater reliability for the detection of advanced lesions was generally substantial for all diagnostic methods.

**Table 4:** Intra- and inter-rater reliability of the different diagnostic methods (radiographic examination: XR, visual-tactile assessment: VT, laser-fluorescence: LF)

	Intra-rater reliability		Inter-rater reliability	
	Any lesions	Advanced lesions	Any lesions	Advanced lesions
XR	1.0	0.67	0.42	0.52
VT	0.66	0.62	0.60	0.65
LF	0.68	0.56	0.72	0.70

## Discussion

Proximal caries assessment is challenging and an accurate diagnosis can oftentimes only be achieved by including additional diagnostic tools. Evidence on the diagnostic quality for these tools, however, is mainly available for coronally located lesions, whereas evidence on proximal root caries lesions is lacking. To our knowledge, this study is the first one evaluating the diagnostic performance of different diagnostic methods for the assessment of primary proximal root caries lesions. We found LF and VT to be more accurate than XR for the detection of advanced root caries lesions. In the detection of any root caries lesions (initial and advanced), LF had the highest accuracy with a relatively small difference to VT, although significant. Intrarater and interrater reliability showed moderate to perfect reproducibility depending on the diagnostic method. As we found significant differences in the diagnostic quality between the different approaches, our null hypothesis was rejected.

Since the root surfaces on the NILT images were overexposed, no evaluable images could be generated. Thus, the manufacturer’s statement that near-infrared transillumination is not suitable for evaluating root caries lesions could be confirmed within the present study. As near-infrared transillumination appears particularly promising for proximal caries [17–19], the purpose of this study was to verify this manufacturer’s statement. The model used here has already been used to investigate the diagnostic quality of proximal secondary caries detection using NILT [12] and therefore appears to be generally suitable in principle for this method.

For the detection of any root caries lesions, the sensitivity of XR and LF was equally high and superior to VT. However, the specificity for XR was lowest among the different assessment methods and the resulting AUC was significantly lower than the AUC of LF and VT. Another in vitro study assessing the diagnostic quality of bitewing radiographs for detection of secondary root caries adjacent to composite restorations found an even lower specificity and sensitivity [20]. Contrarily, another study found for secondary root caries lesions adjacent to amalgam restorations a markedly lower sensitivity but higher specificity [21]. It can be speculated that concavities of the roots or a burn-out effect in the cervical region of the teeth on the radiographs could have led to an increase in false positive findings for XR [20]. The benefit of an additional radiographic examination to VT assessment, which is still considered “gold standard” for proximal caries detection in dental practice, seems therefore to be limited for proximal root caries lesions.

In contrast, LF had the highest specificity and the highest AUC value among the diagnostic approaches tested. The high diagnostic value of LF for root caries assessment can be clinically confirmed by two other studies, in which well accessible root surfaces were evaluated using DIAGNOdent [10, 11]. However, the difference between LF and VT in our study was relatively small, which raises the question whether an additional LF examination for VT diagnosis is generally beneficial. In light of our results, that the diagnostic accuracy of XR was inferior to VT and LF, it would at least be reasonable to use LF rather than XR as an additional diagnostic aid during a visual tactile examination in patients with suspected proximal root caries lesions. In a clinical setting, however, sensitivity and specificity of proximal coronal caries detection with LF might be lower [17, 22]. A potential reason for this could be that the localization of coronal caries lesions in the proximal contact area impedes penetration of the LF probe. The more apically located proximal root caries lesions may allow for a better penetration of the LF probe due to wider interdental spaces. Further studies should evaluate clinically whether proximal root caries diagnosis is facilitated by a better access of the LF probe.

For the detection of advanced lesions, LF and VT had a significantly higher accuracy than XR. Furthermore, LF had the highest sensitivity, but also the lowest specificity among the diagnostic tests. The specificity of LF for detecting any root caries lesions was higher than for detecting advanced lesions. This is in contrast to a systematic review that evaluated LF for coronal caries diagnosis and found a tendency for higher sensitivity and specificity values when carious lesions were already at an advanced stage [23]. The reason for the overestimation of advanced root caries lesions could be that we used the same DIAGNOdent cutoff values for the LF measurements as the manufacturer claims for the investigation of coronal proximal caries. Due to the more direct access to the wider open proximal spaces (see above), higher LF values may have been measured for the root caries lesions than would have been measured for similarly severe caries lesions in the coronal proximal region. Therefore, the cutoff values for the application of LF for root caries diagnostic should be adjusted.

XR had a high specificity, but a low sensitivity for the detection of advanced root caries lesions. It is known that the actual extent of caries lesions is often underestimated on radiographs [24]. For coronal proximal caries, it has also been shown that the sensitivity of caries diagnosis with radiographs decreases with increasing lesion extent [25]. This also seems to be true for proximal root caries lesions, so it can be concluded that radiographs are less suitable as a method for diagnostic screening for proximal root caries lesions.

The diagnostic profile for the detection of advanced caries lesions of VT was similar to that of XR. Both had comparatively low sensitivity but high specificity. In contrast, the specificity of LF for detection of advanced lesions was relatively low, but the sensitivity was distinctly higher than XR and VT. Therefore, and also due to the superior diagnostic performance for the detection of any root caries lesions, LF seems to be a more suitable as a screening method for the detection of root caries than XR and VT. However, the use of the DIAGNOdent pen as a means of screening is associated with increased time and effort compared to VT. Therefore, it is less likely that this method will become established as a standard screening procedure for root caries diagnostic. It is possible, however, that newer 3D scanners or camera systems that integrate laser fluorescence measurement for caries diagnosis will further advance the applicability of LF as a standard diagnostic procedure.

Since the present study is an in vitro investigation, the results are not directly transferable for clinical recommendations. To the best of the authors’ knowledge, no clinical studies are currently available that have investigated the diagnostic quality of all the approaches used here in proximal root caries lesions. However, two clinical studies assessed the diagnostic accuracy of LF in well-accessible root caries lesions with VT as reference test [10, 11]. Both studies reported a sensitivity of 0.80 for LF, which is quite similar to the here found sensitivity for detecting any root caries lesions (0.81). The specificity in these clinical studies, however, was lower (0.80) compared to the specificity for LF found in our study (0.95). Since visual tactile examination as a reference in clinical studies may be less precise than measuring lesion extent on histologic sections, the data from these two clinical studies are only limitedly comparable with the data from the present study. In addition, LF appears to have a higher overall diagnostic accuracy under in vitro conditions than in clinical studies [26]. For the other diagnostic methods tested here, no clinical data are available for testing accuracy in root caries lesions. The lack of clinical evidence indicates that there is a high need for clinical studies addressing different approaches root caries diagnostics [9].

A number of methodological aspects and limitations of this study should be discussed. First, several limitations arise due to the use of an in vitro approach. We used a simple diagnostic model that theoretically should allow the application of different test procedures. Clinical conditions such as saliva exposure or dental plaque and calculus, which can interfere with caries diagnosis, especially when using laser fluorescence, were not simulated in our model. Therefore, the results of our study should be corroborated in a clinical setting. Our in vitro model has been successfully used for the evaluation of NILT in the diagnosis of secondary caries [12]; however, the proximal root caries lesions evaluated here could not be assessed using this approach due to overexposure of the images. It can therefore be assumed that this method is unsuitable for the examination of root caries lesions, as also stated by the manufacturer. Second, we used histological assessment of the lesions as a reference test. While this is common for in vitro evaluation of approaches for caries assessment [27], histologically proven lesions do not necessarily imply the presence of active, and treatment-requiring, caries. However, advanced carious lesions are likely to be active if located proximally due to reduced cleaning ability and should therefore be treated. Third, we assessed proximal root caries lesions at two cutoff points (any and advanced root caries lesions) and assigned the outcome values of the different test methods to these two cutoff points. The distinction as to whether a root caries lesion is present at all or whether it is already in an advanced stage is of clinical importance, as it influences the treatment decision. However, since we used cutoff values for LF that were specified by the manufacturer for the evaluation of coronal proximal caries, this may have led to inaccuracies in this group. An adjustment of the manufacturer’s cutoff values for the detection of proximal root caries is therefore necessary, as it could even improve the diagnostic quality of LF. Last, our study used a convenient sample of teeth with a high prevalence of lesions that may not be representative for most population groups. Moreover, the sample size of our study was not determined by an a priori assumed effect and a resulting sample size calculation but was guided by previous studies in this field [8]. Overall, our sample size was sufficient to find relevant differences and to draw conclusions regarding the comparative accuracy of the different tests.

## Conclusion

Within the limitations of this in vitro study, LF and VT appear to be more accurate than XR for detecting proximal root caries lesions. For initial lesion stages in particular, LF appears to be more accurate than VT. NILT is not suitable for proximal root caries diagnosis.

## Supplementary Information

Below is the link to the electronic supplementary material.

Supplementary file1 (DOCX 7.36 MB)

## References

[1] Kassebaum NJ, Bernabe E, Dahiya M, Bhandari B, Murray CJ, Marcenes W (2015). Global burden of untreated caries: a systematic review and metaregression. J Dent Res.

[2] Schwendicke F, Krois J, Schiffner U, Micheelis W, Jordan RA (2018). Root caries experience in Germany 1997 to 2014: analysis of trends and identification of risk factors. J Dent.

[3] White D, Pitts N, Steele J, Sadler K, Chadwick B (2011) Disease and related disorders - a report from the Adult Dental Health Survey 2009. Adult Dental Health Survey 2009-summary Report and Thematic Series. 1–55

[4] Bots-VantSpijker PC, Vanobbergen JN, Schols JM, Schaub RM, Bots CP, de Baat C (2014). Barriers of delivering oral health care to older people experienced by dentists: a systematic literature review. Community Dent Oral Epidemiol.

[5] Lo EC, Luo Y, Tan HP, Dyson JE, Corbet EF (2006). ART and conventional root restorations in elders after 12 months. J Dent Res.

[6] Wierichs RJ, Meyer-Lueckel H (2015). Systematic review on noninvasive treatment of root caries lesions. J Dent Res.

[7] Kapor S, Rankovic MJ, Khazaei Y, Crispin A, Schuler I, Krause F (2021). Systematic review and meta-analysis of diagnostic methods for occlusal surface caries. Clin Oral Investig.

[8] Janjic Rankovic M, Kapor S, Khazaei Y, Crispin A, Schuler I, Krause F (2021). Systematic review and meta-analysis of diagnostic studies of proximal surface caries. Clin Oral Investig.

[9] Fee PA, Macey R, Walsh T, Clarkson JE, Ricketts D (2020). Tests to detect and inform the diagnosis of root caries. Cochrane Database Syst Rev.

[10] Zhang W, McGrath C, Lo ECM (2016). Effectiveness of DIAGNOdent in detecting root caries without dental scaling among community-dwelling elderly. Oral Health Prev Dent.

[11] Zhang W, McGrath C, Lo EC (2009). A comparison of root caries diagnosis based on visual-tactile criteria and DIAGNOdent in vivo. J Dent.

[12] Elhennawy K, Askar H, Jost-Brinkmann PG, Reda S, Al-Abdi A, Paris S (2018). In vitro performance of the DIAGNOcam for detecting proximal carious lesions adjacent to composite restorations. J Dent.

[13] Topping GVA, Pitts NB (2009). Clinical visual caries detection. Monogr Oral Sci.

[14] Umwali A, Askar H, Paris S, Schwendicke F (2016). Radiographic, antibacterial and bond-strength effects of radiopaque caries tagging. Sci Rep.

[15] Karlsson L, Johansson E, Tranaeus S (2009). Validity and reliability of laser-induced fluorescence measurements on carious root surfaces in vitro. Caries Res.

[16] Landis JR, Koch GG (1977). The measurement of observer agreement for categorical data. Biometrics.

[17] Kocak N, Cengiz-Yanardag E (2020). Clinical performance of clinical-visual examination, digital bitewing radiography, laser fluorescence, and near-infrared light transillumination for detection of non-cavitated proximal enamel and dentin caries. Lasers Med Sci.

[18] Abogazalah N, Eckert GJ, Ando M (2017). In vitro performance of near infrared light transillumination at 780-nm and digital radiography for detection of non-cavitated approximal caries. J Dent.

[19] Schwendicke F, Elhennawy K, Paris S, Friebertshauser P, Krois J (2020). Deep learning for caries lesion detection in near-infrared light transillumination images: A pilot study. J Dent.

[20] Rodrigues JA, Neuhaus KW, Hug I, Stich H, Seemann R, Lussi A (2010). In vitro detection of secondary caries associated with composite restorations on approximal surfaces using laser fluorescence. Oper Dent.

[21] Neuhaus KW, Rodrigues JA, Seemann R, Lussi A (2012). Detection of proximal secondary caries at cervical class II-amalgam restoration margins in vitro. J Dent.

[22] Ozkan G, Guzel KGU (2017). Clinical evaluation of near-infrared light transillumination in approximal dentin caries detection. Lasers Med Sci.

[23] Gimenez T, Braga MM, Raggio DP, Deery C, Ricketts DN, Mendes FM (2013). Fluorescence-based methods for detecting caries lesions: systematic review, meta-analysis and sources of heterogeneity. PLoS ONE.

[24] Syriopoulos K, Sanderink GC, Velders XL, van der Stelt PF (2000). Radiographic detection of approximal caries: a comparison of dental films and digital imaging systems. Dentomaxillofac Radiol.

[25] Ko HY, Kang SM, Kim HE, Kwon HK, Kim BI (2015). Validation of quantitative light-induced fluorescence-digital (QLF-D) for the detection of approximal caries in vitro. J Dent.

[26] de Paula AB, Campos JA, Diniz MB, Hebling J, Rodrigues JA (2011). In situ and in vitro comparison of laser fluorescence with visual inspection in detecting occlusal caries lesions. Lasers Med Sci.

[27] Brouwer F, Askar H, Paris S, Schwendicke F (2016). Detecting secondary caries lesions: a systematic review and meta-analysis. J Dent Res.

