# Supplementary material for: Accuracy of different approaches for detecting proximal root caries lesions in vitro
Source: Clin Oral Investig. 2022 Sep 16;27(3):1143–51. doi: 10.1007/s00784-022-04709-1 (PMC9985551; doi:10.1007/s00784-022-04709-1)
Supplement: Supplementary file 1 — Supplementary file1 (DOCX 7.36 MB) [file 784_2022_4709_MOESM1_ESM.docx]

# Appendix

| a)  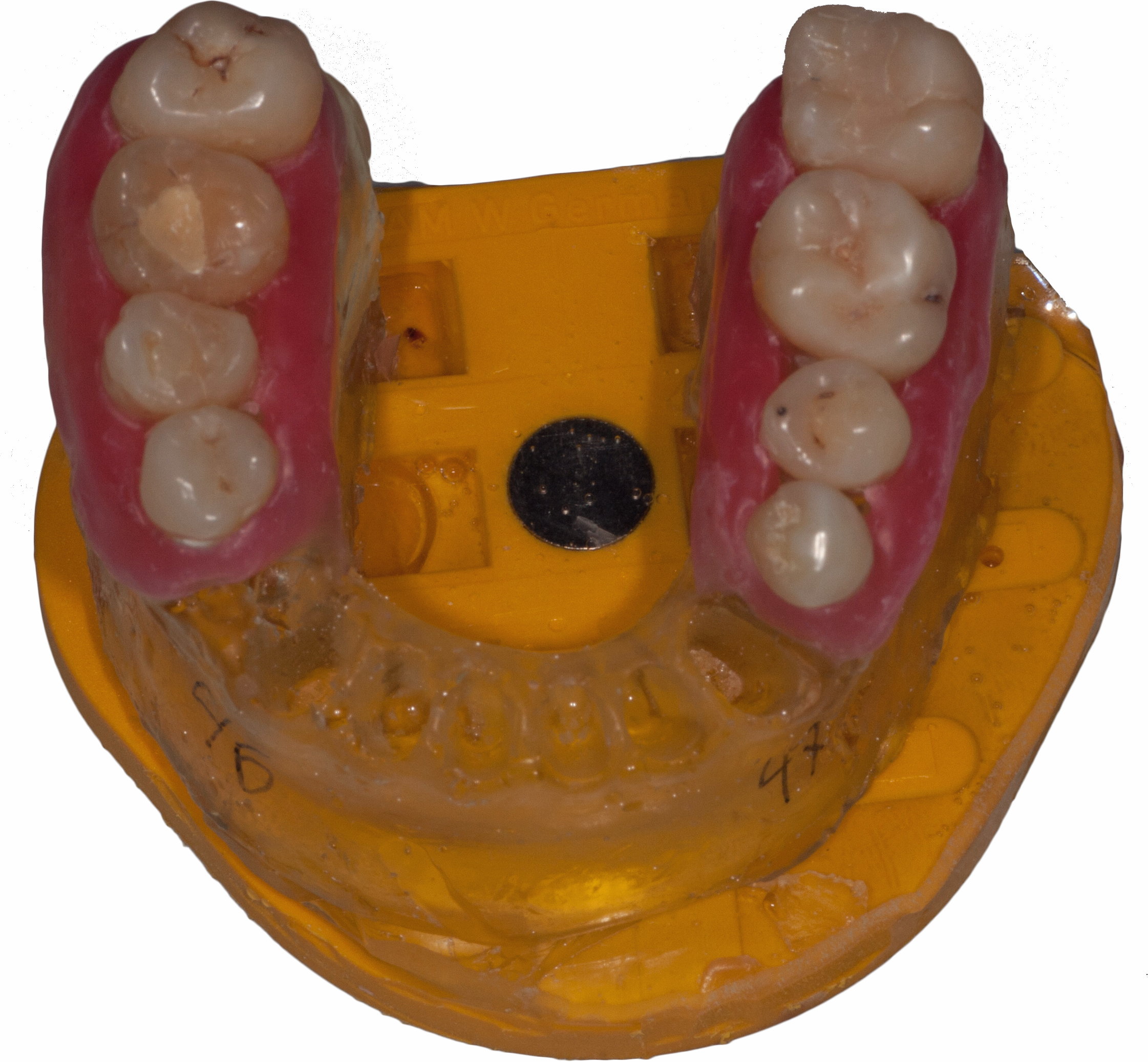 | b) 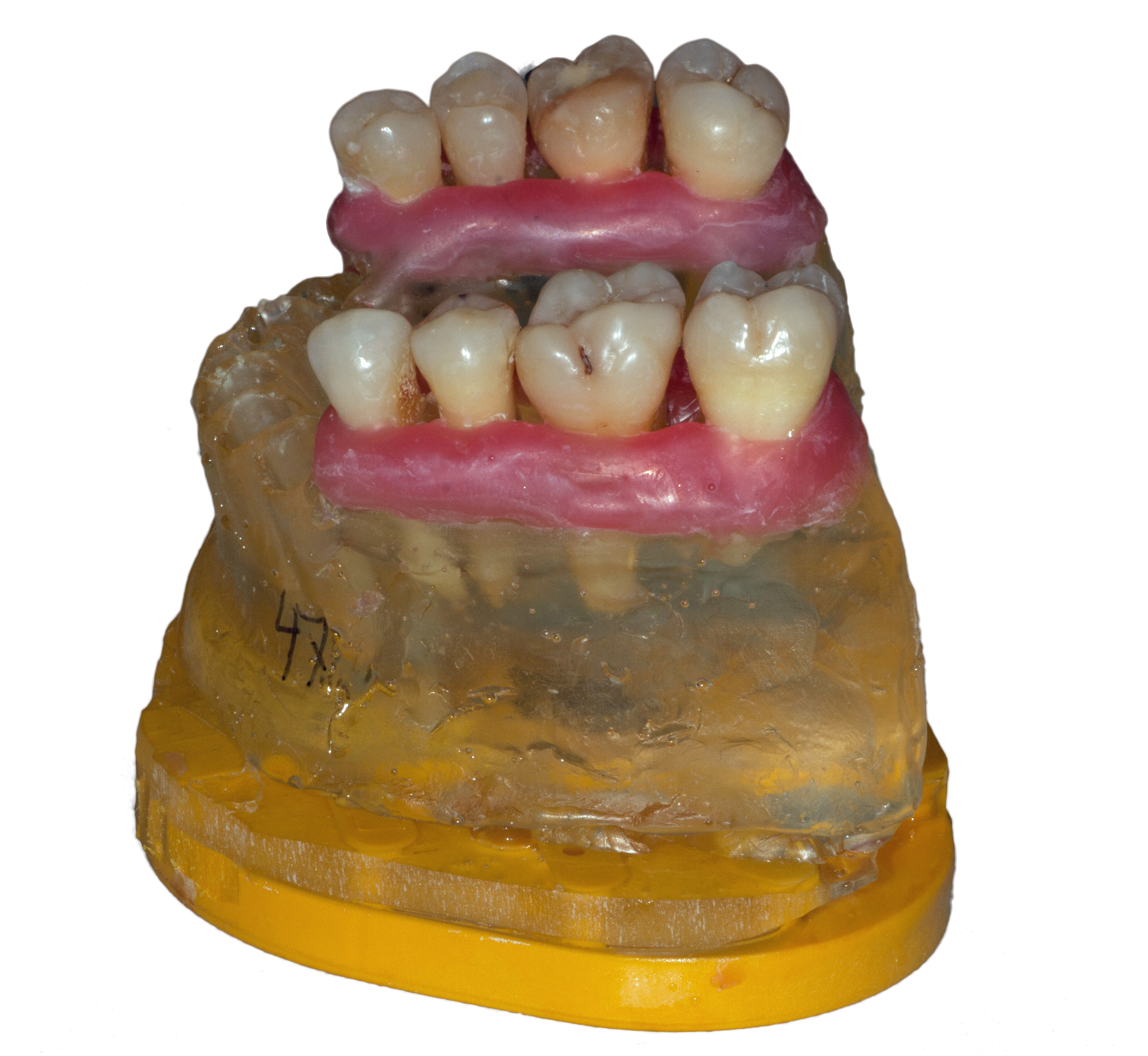 |
| --- | --- |

**Figure S1** Model used for evaluation of the different diagnostic approaches from occlusal (a) and buccal (b).


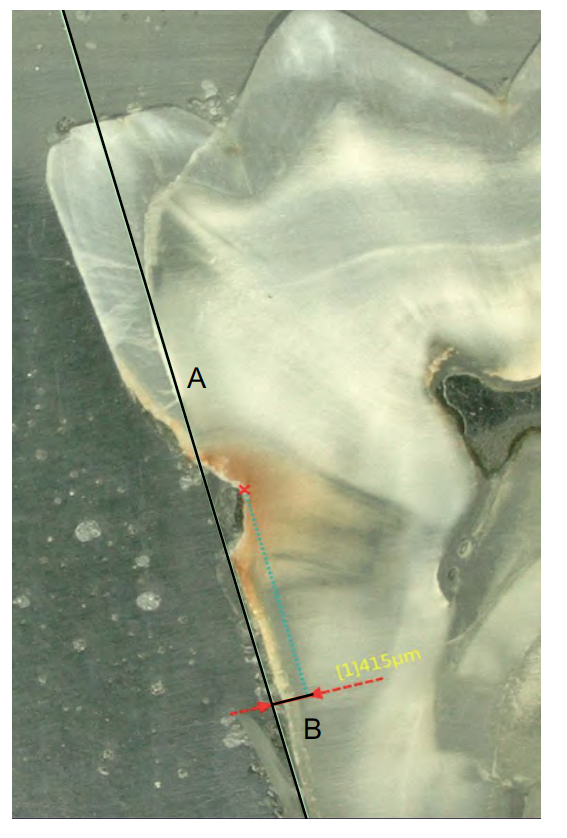


**Figure S2** Determination of the lesion extension as a reference test. The Image of the tooth section was obtained at 20x magnification under the digital microscope. The line A was drawn along the putative original root surface. Line B is perpendicular to line A and represents the distance between line A and the deepest point of the cavity (here: 415µm).


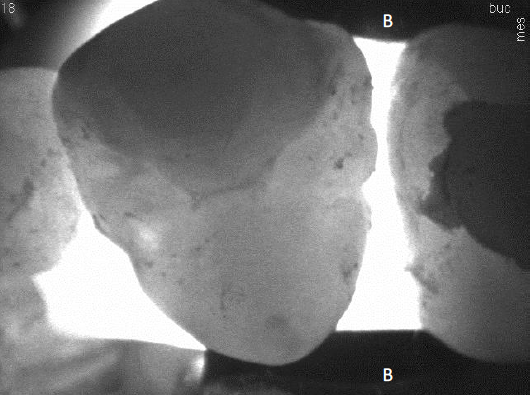


**Figure S3** Exemplary NILT image. The interdental space is overexposed - an assessment of the root surface is not possible. Abbreviations: B: Illumination arms, mes: mesial direction, buc: buccal direction.
